# Synchronous solid pseudopapillary neoplasm of the pancreas with intrahepatic cholangiocarcinoma in a young male patient: An unusual deadly occurrence

**DOI:** 10.1016/j.ijscr.2021.105841

**Published:** 2021-03-26

**Authors:** Moshawa Calvin Khaba, Nkomba Christopher Kalenga, Ramatsimele Rebothile Phetla, Victor Mngomezulu, Moses Aschenaz Balabyeki

**Affiliations:** a Department of Anatomical Pathology, Dr George Mukhari Academic Laboratory, National Health Laboratory Services, Sefako Makgatho Health Sciences University, South Africa; b Department of General Surgery, Hepatopancreatobiliary Unit, Dr George Mukhari Academic Hospital, Sefako Makgatho Health Sciences University, South Africa; c Department of Diagnostic Radiology, Chris Hani Baragwanath Academic Hospital, University of the Witwatersrand, South Africa

**Keywords:** Solid pseudopapillary neoplasm, Intrahepatic cholangiocarcinoma, Young, African, Male

## Abstract

**Introduction and importance:**

Solid pseudopapillary neoplasm of the pancreas (SPN) is a tumour with low malignant potential, albeit with good prognosis. Intrahepatic cholangiocarcinoma (iCCA) is a malignant and aggressive tumour with poor prognosis.

**Clinical presentation:**

We report a case of a 38 years old African male patient with abdominal pain for 5 years that worsened in the preceding 5 months. Radiology showed pancreatic and liver lesions which were thought to be malignant and benign, respectively. However, intra-operative and histopathological assessment confirmed SPN and iCCA which were contrary to radiological findings. Whilst surgery was uneventful, the patient died after 15 days.

**Clinical discussion:**

Whilst SPN is commonly seen in young females, it is rare in males and has been associated with aggressive behaviour. The prognosis is good, albeit the presence of metastasis. iCCA is rare in younger population notwithstanding the presence of risk factor. Combination of iCCA and SPT has not been described. Both these tumours do not share risk factors, pathogenesis or molecular alterations.

**Conclusion:**

The concomitant occurrence of these two pathologies in young male patient is unusual and preoperative diagnosis may be very difficult.

## Highlights

• Solid pseudopapillary neoplasm of the pancreas is rare in males.
• Intrahepatic cholangiocarcinoma is not uncommon in the African population, however is rare in younger population.
• According to the literature search, this is the first case of synchronous solid pseudopapillary neoplasm of the pancreas and intrahepatic cholangiocarcinoma.
• Multidisciplinary team is important in management of cases with clinical conundrum.

## 1 Introduction

Solid pseudopapillary neoplasm of the pancreas (SPN) is a tumour of low malignant potential with good prognosis and accounts for 1–2% of all exocrine pancreatic tumours [1]. Invasion of adjacent organs or distant metastasis may happen with the liver as the common site of metastasis [2,3]. Even in the presence of disseminated disease, the 5-year survival rate is estimated to be approximately 95%–97% [4,5].

Intrahepatic cholangiocarcinoma (iCCA) is a rare and aggressive epithelial tumour. It is the second most common cancer arising from the liver. It accounts for 3% of all gastrointestinal cancers and 10% of all cholangiocarcinomas [6].

Herein, we report a case of a male patient with SPN and iCCA with overlapping clinical features and distinct radio-pathological features. This dual pathology combination is unusual and necessitates a literature review.

This case has been reported in line with the SCARE criteria [7].

## 2 Case presentation

38 years old male African patient who presented with a 5 years history of abdominal pain that worsened during the preceding 5 months. The pain was associated with vomiting, early satiety, loss of appetite and significant weight loss. He did not have any comorbidities nor social habits. On general examination, he was emaciated with non-tender hepatomegaly. Other systems were unremarkable. The laboratory investigations were also unremarkable.

**Fig. 1:**
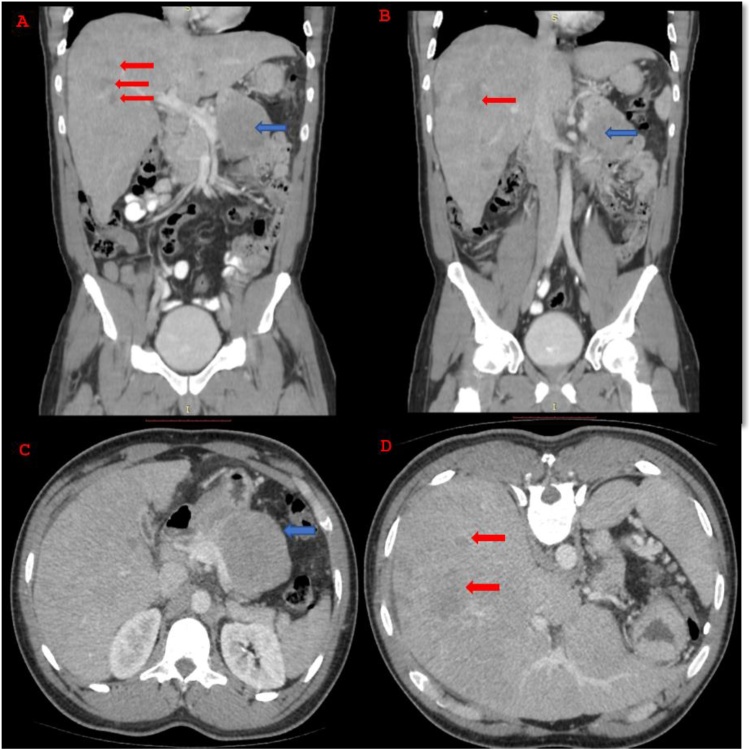
Abdominal CT Images. **A** and **B**: Coronal images: Hepatomegaly with vague rim-enhancement of the hepatic lesions (red arrows). The complex cystic lesion in the pancreas (blue arrow). No suspicious metastatic bony lesions were visualised. **C** and **D**: Axial plane (contrast): **C**, well-defined hypodense lesion (blue arrow) in the pancreatic body; **D**, poorly circumscribed hypodense liver lesions of variable sizes throughout the liver parenchyma (red arrow) that do not follow any particular enhancement pattern. There is no dilatation of the biliary ducts.

CT scan of the abdomen showed a hypodense mass in the body of the pancreas measuring 66 × 69 × 63 mm. It abutted the splenic vein with no clear fat plane in between them. There was a clear plane of separation between the mass and the coeliac trunk and its major branches. The pancreatic duct in the body and the tail, distal to the mass, was prominent and measured 5,4 mm. Whilst pancreatic neuroendocrine tumour was favoured at this point, other considerations included lymphoma or SPN. Moreover, there was hepatomegaly with multiple focal liver lesions. They showed progressive fill in and were predominantly iso-attenuating on the delay sequences. There were no dilated intrahepatic bile ducts dilatation (Fig. 1). In conjuction with the ultrasound, the impression was that of a haemangioma.

At this point, the possibilities were that the patient has two separate pathologies in the liver and pancreas, or the pancreatic lesion has metastasised to the liver. Once the patient was optimised for surgery, a distal pancreatectomy with splenectomy was performed. Intra-operatively, there were multiple firm liver lesions which did not look like haemangioma; therefore, a liver biopsy was taken. The surgery was uneventful. Nonetheless, on the 8th day post-surgery, he started to deteriorate and died on the 15th day.

**Fig. 2:**
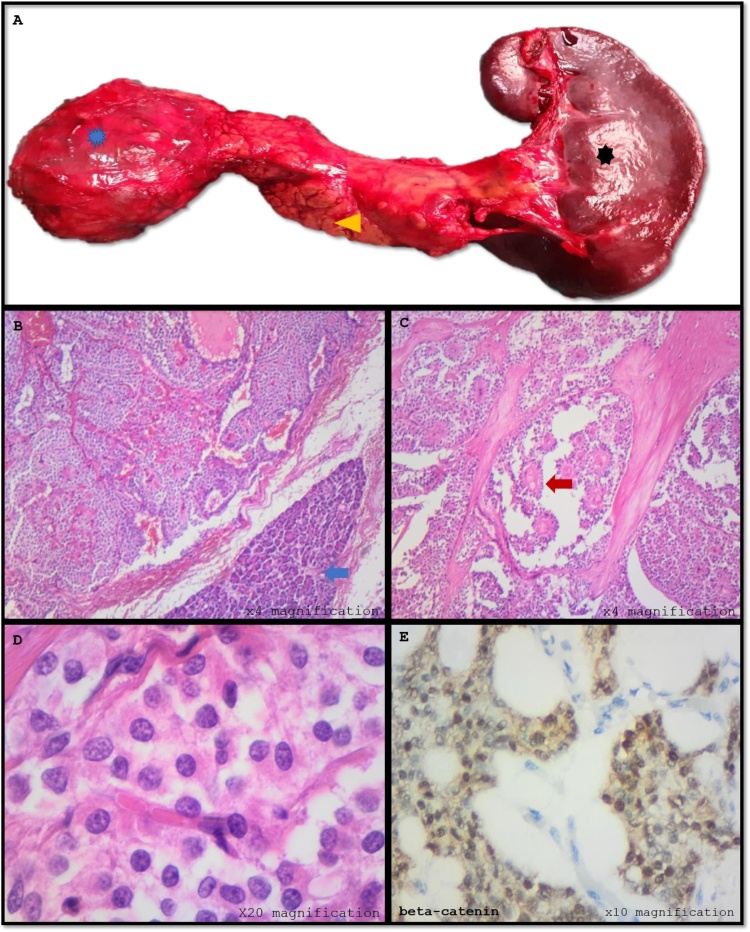
Macroscopic and microscopic photographs of solid pseudopapillary tumour of pancreas. **A:** Fresh pancreas() with body tumour () and normal spleen (); **B & C:** pancreatic tissue () with tumour arranged in nests and pseudopapillae (); **D:** uniform cells with granular cytoplasm and bland nuclei; **E:** positive nuclear beta-catenin immunostain.

For histopathological assessment, we received a pancreas measuring 150 × 65 × 40 mm and weighing 220 g with a tumour located on the body that measured 65 × 80 × 60 mm. On cut section, it was circumscribed, firm and white-tan with haemorrhagic areas. The accompanying spleen appeared normal (Fig. 2A).

A separate piece of liver tissue measuring 10 × 10 × 5 mm was also received.

Microscopic examination of the pancreas showed SPN evidenced by well circumscribed tumour arranged in nests, tubules and pseudopapillae (Fig. 2B and C). The tumour cells had moderate pale and finely granular cytoplasm with uniform nuclei and conspicuous nucleoli (Fig. 2B–D). Cytological atypia, necrosis or atypical mitosis were not seen. The tumour cells were positive for CD56, synaptophysin, CD10 and beta-catenin (Fig. 2E). AE1/AE3, CK7 and chromogranin were negative.

**Fig. 3:**
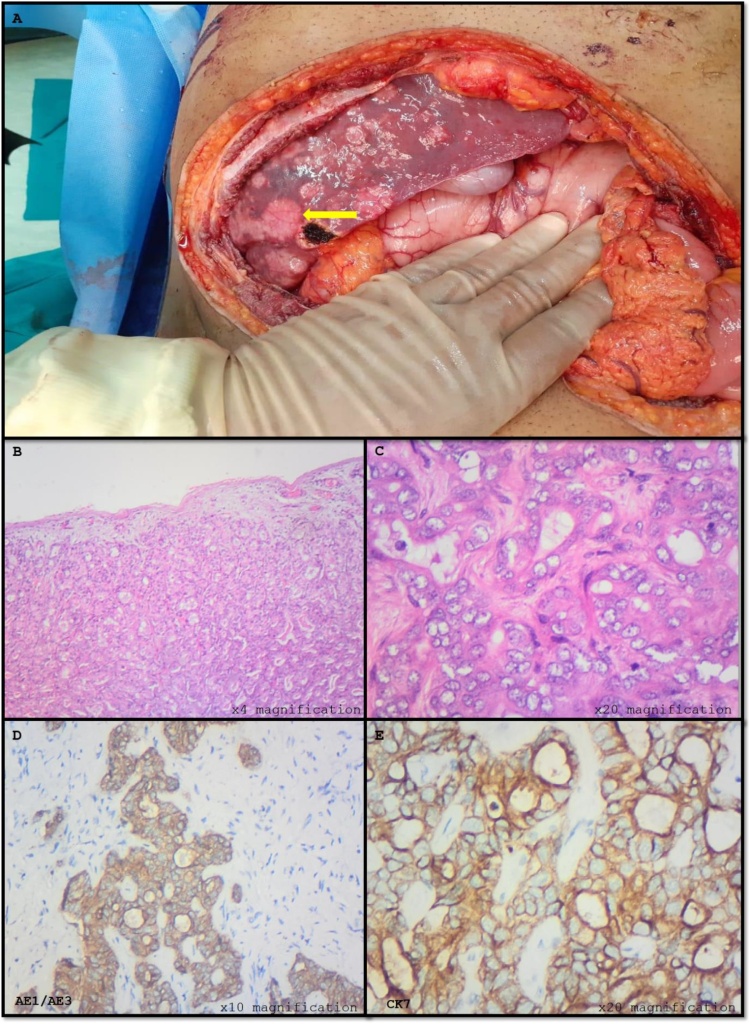
Intrahepatic cholangiocarcinoma. **A:** Intraoperative image of the liver with multiple white-tan tumour nodules (); **B**: Malignant tumour arranged in glands (low magnification); **C**: Tumour cells with pleomorphic nuclei and mitosis; **D**: Positive AE1/AE3; **E**: Positive cytokeratin 7(CK7).

Microscopic examination of the liver confirmed intrahepatic cholangiocarcinoma (iCCA) evidenced by an infiltrating tumour arranged in glands within a desmoplastic stroma (Fig. 3A & B). The tumour cells were large with eosinophilic cytoplasm, pleomorphic and vesicular nuclei with prominent nucleoli (Fig. 3B and C). The tumour cells were positive for AE1/AE3 and CK7 (Fig. 3D and E). Beta-catenin, CD10 and neuroendocrine markers were negative.

## 3 Discussion

Clinicopathological criteria for malignancy in SPN have not yet been established. However, lymph node metastasis and local invasion are regarded as histological evidence of malignancy [2,8,5]. The clinical features associated with poor prognosis or malignancy include older age at presentation, males and tumour metastasis at the first operation. The histopathological features include perineural and angioinvasion, high mitotic rate, spindling of tumour cells, anaplastic giant cells, capsular invasion and necrosis [2,[8], [9], [10]].

Whilst the Ki67 index ≥4% has been reported to show poor recurrence-free, its role is still debateable [8,11].

Some studies have associated focal capsular disruption with higher risk for malignancy [2,5,9].

En bloc resection with clear margins is the treatment of choice and its failure increases the risk of recurrence [2,9]. Apart from the index patients being a male which is associated with aggressive behaviour, there were no clinicopathological features associated with poor prognosis.

Cholangiocarcinoma (CCA) is classified into intrahepatic (iCCA), perihilar (pCCA) and distal (dCCA) CCA based on anatomical site and iCCA is the least common [6,[12], [13], [14]].

The incidence peak is at seventh decade with slight male predominance. It rarely occurs in individual less than 40 years of age [6,14,15].

Geographical regions reflect differences in environmental, genetic, environmental and cultural inclination to this malignancy [12]. iCCA is commonly seen in the Eastern Europe. Although it may be sporadic in most cases, it is associated with primary sclerosing cholangitis, toxins, hepatobiliary flukes, congenital cysts and hepatolithiasis [[12], [13], [14]].

The index patient did not have any of predisposing factor for iCCA and he was of African origin. This, combined with non-specific clinical features, may have contributed in delayed clinical diagnosis.

Diagnostic imaging and a high degree of clinical suspicion play a critical role in timely diagnosis, staging, and evaluation for surgical resectability [14].

Ultrasound (U/S) is used as the first radiological modality for assessment of iCCA. It has a sensitivity and specificity of 89 and 95%, respectively. It shows hypoechoic lesion with or without ductal dilatation. Contrast agents improve its diagnostic accuracy. Once a suspicious lesion is seen on U/S, further imaging studies are mandatory [6,13,14].

Whilst only CT scan was performed on the index case, MRI is the image modality of choice. It has high sensitivity and specificity compared to CT scan.

MRI shows hypo-intense on T1-weighted and hyper-intense on T2-weighted images. The arterial phase show peripheral enhancement followed by progressive and concentric filling-in of the tumour with contrast material. Pooling of contrast on delayed images is indicative of fibrosis and may be suggestive of an iCCA in the right clinical setting [12,14].

The histopathological diagnosis of iCCA shows an adenocarcinoma tubular and/or papillary growth pattern with a fibrous background. Before a definite diagnosis is made, extra-hepatic metastasis and hetaocellular carcinoma (HCC) need to be excluded. This requires negative immunohistochemical stains such as TTF1 (lung), CDX2 (colon, stomach, pancreas), Hep-Par-1 and glypican 3 (HCC). iCAA is positive for CK7 and CEA, and negative for CK20 [13,15,16].

Complete surgical resection remains the only option for cure with an estimated median survival ranging from 27 to 36 months [6,13].

The overall prognosis is poor. Patients with non-resectable disease at presentation die within 6–12 months from diagnosis. The survival rate is low and less than 5% of patients can still be alive after 5 years. 75% of patients die between the first year from diagnosis [13].

## 4 Conclusion

The presence of multiple primary tumours is a known concept in medicine for decades with various combinations reported. This case highlights the importance of multi-disciplinary team and collaboration of surgeons, radiologists, histopathologists and oncologist in optimal management of cases with clinical challenges.

## Declaration of Competing Interest

None.

## Sources of funding

None.

## Ethical approval

Sefako Makgatho University Research Ethics Committee(SMUREC) approved the publication of this case report.

SMUREC/M/339/2020.

## Consent

Written informed consent was not obtained from the patient. The head of our medical team has taken responsibility that exhaustive attempts have been made to contact the family and that the paper has been sufficiently anonymised not to cause harm to the patient or their family. A copy of a signed document stating this is available for review by the Editor-in-Chief of this journal on request.

## Author contribution

All authors wrote the case report. Dr MC Khaba organized the manuscript and critically revised the paper.

## Registration of research studies

Not applicable.

## Guarantor

Dr MC Khaba.

## Provenance and peer review

Not commissioned, externally peer-reviewed.
